# P-1744. Clinical Characteristics and Prognostic Factors of Invasive Fungal Disease Caused by Rare Fungi in A University Hospital in Thailand

**DOI:** 10.1093/ofid/ofaf695.1915

**Published:** 2026-01-11

**Authors:** Pacharasupon Khiawhom, Piriyaporn Chongtrakool, Methee Chayakulkeeree

**Affiliations:** Faculty of Medicine Siriraj Hospital, Mahidol University, Bangkoknoi, Krung Thep, Thailand; Faculty of Medicine Siriraj Hospital, Mahidol University, Bangkoknoi, Krung Thep, Thailand; Faculty of Medicine Siriraj Hospital, Mahidol University, Bangkoknoi, Krung Thep, Thailand

## Abstract

**Background:**

Invasive fungal diseases (IFDs) caused by rare species present significant challenges, particularly in Thailand, where data are limited. This study aims to enhance understanding and raise awareness by investigating the risk factors, and clinical outcomes of patients with rare IFDs in Thailand.

**Methods:**

This retrospective observational study reviewed the medical records of 163 patients diagnosed with IFDs, excluding aspergillosis, cryptococcosis, and candidiasis, at Siriraj Hospital, Thailand, between 2005 and 2022. Baseline characteristics, risk factors, and clinical outcomes were analyzed.

**Results:**

Among the 163 patients, the mean age was 56.8 years, with 57.1% male. Common underlying conditions included diabetes mellitus (39.9%), hematologic malignancies (20.1%), and organ transplantation (11.0%). Notable risk factors included steroid use (11%) and neutropenia (6.7%). Blood cultures were positive in 30.1%. The predominant syndromes were disseminated infection (90.8%) and pulmonary mycoses (22.1%), with cutaneous involvement in 32.5%. The mortality rate was 38%. Multivariate analysis revealed that ICU admission (aOR 6.7, *p* = 0.002), endotracheal intubation (aOR 6.0, *p* = 0.002), and hematologic malignancies (aOR 3.6, *p* = 0.015) independently associated with mortality. *Fusarium* (30.1%) was the most common rare mold, followed by *Rhizopus* (12.3%), and *Scedosporium* (5.5%). Hyalohyphomycosis accounted for 63 cases. Fungemia occurred in 20.6% of fusariosis cases, with a mortality rate of 32.7%, while *Acremonium* infections had a mortality rate of 25%. In hyalohyphomycosis, mortality linked to endotracheal intubation (aOR 10.8, *p* = 0.002) and hematologic malignancies (aOR 8.6, *p* = 0.006). *Trichosporon* and *Apiotrichum* were the most common rare yeasts, identified in 50 cases, with a mortality rate of 46%. Fungemia was present in 56%. ICU admission was a strong risk factor for mortality (aOR 38.0, *p* < 0.001).

**Conclusion:**

Rare IFDs, particularly those caused by *Fusarium* and *Trichosporon*, are emerging concerns. Independent factors associated with mortality include ICU admission, endotracheal intubation, and hematologic malignancies. Early recognition and targeted management are essential for improving patient outcomes.

**Disclosures:**

All Authors: No reported disclosures

